# Elevated autistic features in Parkinson’s disease and other motor disorders

**DOI:** 10.1177/13623613251362267

**Published:** 2025-08-26

**Authors:** Ipsita Dey, Swarnima Pathak, Sreerupa Chakrabarty, Matthew K. Belmonte, Supriyo Choudhury, Hrishikesh Kumar, Bhismadev Chakrabarti

**Affiliations:** 1 India Autism Center, India; 2 The Com DEALL Trust, India; 3 Department of Psychology, Nottingham Trent University, UK; 4 Department of Neurology, Institute of Neurosciences Kolkata, India; 5 Department of Psychology, Ashoka University, India; 6 Centre for Autism, School of Psychology and Clinical Language Sciences, University of Reading, UK

**Keywords:** autism spectrum disorders, global, medical comorbidity, motor disorders, neurological conditions, Parkinson’s disease

## Abstract

Biological accounts have suggested an overlap between Parkinson’s disease and autism despite their being studied largely at opposite ends of the life course. Characterising this overlap can identify potentially shared aetiologies and care pathways for these conditions. However, this overlap has so far only been tested in older autistic adults who show greater Parkinson’s disease traits. The converse has not been directly assayed, that is, if adults with Parkinson’s disease have higher autistic features. This preregistered study addressed this gap in the literature by asking whether adults with Parkinson’s disease manifest elevated autistic traits. To test whether any such overlap might be unique to Parkinson’s disease, we included two control groups: (1) people without any parkinsonism but with motor disability of neurological or neurovascular origin (other motor disorders), and (2) typically ageing controls with no motor disorders. We tested N = 330 participants (equal numbers of Parkinson’s disease, other motor disorders and typically ageing controls) on their autistic traits and cognitive abilities. Clinical diagnoses were verified through a tertiary neurology clinic. Higher autistic traits were noted in both Parkinson’s disease and other motor disorder groups compared to the typically ageing controls, suggesting an association between motor disorders and dimensional autistic traits. Exploratory analyses revealed a clear pattern of results in males, where Parkinson’s disease was associated with the highest autistic traits, followed by the other motor disorders, and then by the typically ageing group. No such pattern was observed in females. These results are not explained by differences in language or age or reporter effects. This new evidence suggests a sex-specific overlap between these conditions and highlights the need for accounting for elevated autistic features in planning support for males with Parkinson’s disease and other movement disorders.

**Lay Abstract:**

People with autism are three times more likely than non-autistic people to develop Parkinson’s disease in later life, and some of the same genetic variants contribute to risks for both these conditions. Although Parkinson’s disease is more common in people with autism, is autism correspondingly more common in people with Parkinson’s disease? Or what about autistic patterns of thought and behaviour, even in Parkinson’s patients who are not also diagnosed, or diagnosable, with autism itself? We surveyed such autistic traits in three groups of older people: Parkinson’s patients, patients with other neurological disorders of movement and those without any neurological or movement disorder or condition. Men with Parkinson’s disease and men with non-parkinsonian motor disorders had more autistic traits than normal. Women with Parkinson’s or other motor disorders, on the other hand, did not differ from women without any motor disorder. This was true no matter in which of the three languages surveys were given, and no matter whether it was patients themselves or their caregivers who completed the survey. Some underlying genetic or other biological shared factors might increase autistic traits not only in Parkinson’s disease but also in some of the other motor disorders represented in this study’s comparison group, for example, essential tremor. Conversely, Parkinson’s disease might not be the only motor disorder to which people with autism stand at heightened risk. Assessment of autistic traits should be considered as part of care planning for people with Parkinson’s disease or other motor disorders.

## Introduction

Autism spectrum disorder, henceforth ‘autism’, is a lifelong neurodevelopmental condition. The characteristics of autism usually become evident during early childhood and include a wide range of differences in social communication, patterns of interests and sensory sensitivity/reactivity (American Psychiatric Association, 2013). Despite research indicating that individuals with autism encounter significant health challenges due to accompanying physical conditions, such as epilepsy, obesity and gastrointestinal (GI) issues (Ward et al., 2023), physical health among autistic individuals has received less attention compared to mental health outcomes. Most of the evidence in this area comes from cross-sectional studies and lacks sufficient insight into age-related physical conditions (Rydzewska et al., 2021).

In particular, there is a notable scarcity of research studies focusing on autism and its symptoms in older adults (Happé & Charlton, 2011; Robison, 2019; Sonido et al., 2020; Wright et al., 2016), accounting for less than 1% of published studies on autism. This gap in the literature is arguably due to the neurodevelopmental nature of this condition, as well as the reduced recognition and evolving diagnostic criteria (Russell et al., 2021) of autism and its features in past decades. The consequent dearth of evidence in this domain creates uncertainty for older individuals on the autistic spectrum and their families, communities and healthcare providers regarding how to anticipate and address their evolving needs as they age (Mason et al., 2022). Like other complex neuropsychiatric diagnoses, autism’s categorical diagnosis presents an extreme of a continuum of cognitive, affective, perceptual and motor variation that extends throughout the general population. Where dimensional trait-based rather than categorical diagnostic associations are the objective, studies of such normal population-wide diversity can offer greater variance to be exploited in correlational studies and, thus, greater statistical power than that of a study confined to the categorical diagnosis itself.

One condition that has been suggested to overlap strongly with autism is Parkinson’s disease (PD) and its symptoms (Hollander et al., 2009; Mai et al., 2023). In support of this theoretical suggestion, PD and related disorders have been reported to be three times more prevalent in autistic adults, compared to adults from the general population (Croen et al., 2015). In a more recent study, the prevalence of PD was 6.6% among autistic adults aged 65 years and older, compared to the non-autistic control group, with a prevalence of 1.2% (Hand et al., 2020). Adding to this body of evidence, a large-scale longitudinal study by Yin et al. (2025) has confirmed that individuals with autism have a significantly higher risk of developing PD, even after adjusting for numerous potential confounding factors.

PD is a condition marked by progressive neurodegeneration primarily affecting motor function. Striatal dopaminergic circuits involved in the control of movement are particularly affected, but sensory and prefrontal association areas are also affected to a lesser extent (Braak & Braak, 2000). PD is associated with both motor and cognitive symptoms (Hammond et al., 2007). Such symptoms have also been found to occur more commonly in middle-aged and elderly autistic individuals compared to the general population (Hand et al., 2020). Older autistic adults also demonstrate greater symptoms of parkinsonism via both self-report measures and instrumental and observational assessments (Geurts et al., 2021; Mostert-Kerckhoffs et al., 2019; Starkstein et al., 2015).

Besides the core motor issues, PD is also accompanied by a range of other affective and cognitive impairments, some of which are also observed in the autistic spectrum. Non-demented PD patients often show a degree of neurocognitive impairment accompanied by executive dysfunction (Riedel et al., 2010). Similar challenges with executive function tasks are also commonly observed in autism (Demetriou et al., 2017). Several studies have also reported differences in empathy in adults with PD (Coundouris et al., 2020), similar to those that have been reported in autism (Baron-Cohen & Wheelwright, 2004; Blacher et al., 2003; Gillberg, 1992; Shamay-Tsoory et al., 2002). In reviewing this symptomatic overlap between PD and Autism Spectrum Conditions (ASC). Hollander et al., (2009) reported that both individuals with PD and autism display a range of obsessive-compulsive (OC) and related behaviours. These behaviours are repetitive in nature and appear similar to those observed in many autistic individuals. One such phenomenon is punding, which is found in 1.4% to 14% of PD patients (Evans et al., 2004; Fernandez & Friedman, 1999; Friedman, 1994; Miyasaki et al., 2007). This symptomatic overlap between the two conditions is mirrored by several genetic studies, which have implicated overlapping loci in both PD and ASC (Kim et al., 2020; Mai et al., 2023).

While initial reports have demonstrated greater PD traits in older autistic individuals, the converse has not been directly tested, that is, autistic symptoms have not been investigated in PD patients. It is necessary to examine this possibility in order to test whether shared biological and/or environmental factors increase the likelihood of both PD and autism. For example, if autism-relevant genetic factors increase the likelihood of developing parkinsonism, but PD -related factors do not increase autistic features, it would argue against an explanation based on shared factors. It is also not clear whether the suggested overlap of symptoms between autism and PD is specific to PD or whether it extends to non-Parkinson’s motor disorders. In previous reports discussed above, where similar measures have been investigated, the control group usually comprises neurotypical (NT) adults only (e.g. Fabbri et al., 2018; Narme et al., 2013). To address this gap in the literature, this study aims to examine whether autistic traits are elevated in individuals with PD compared to two matched control groups: (1) individuals with other non-parkinsonian motor disorders (OMD) and (2) typically ageing adults without known motor conditions. If the suggested overlap between autism and PD is specific to PD, then we expect to note a higher degree of autistic symptoms in PD compared to both of these control groups. Through this study, we aim to advance our understanding of the degree and specificity of overlap between these two conditions.

## Methods

The study design was observational, and data were collected at a single time point from the participants and their caregivers. The study received ethical approval from the Institutional Research Ethics Board (IREB) at the Institute of Neuroscience, Kolkata (IN-K). All participants or their legal caregivers provided informed written consent for participation.

## Participants

### Sample

The sample included three groups: (1) Individuals with a confirmed clinical diagnosis of PD following UK Parkinson’s Disease Society Brain Bank criteria for idiopathic PD (Hughes et al., 1992). (2) Individuals without any parkinsonism, but with motor disability of neurological or neurovascular origin (OMD) (see Supplementary Table 3), and (3) NT, that is, individuals without PD or any known motor disabilities.

Participants were recruited from two sources: (1) Both the clinical groups, PD and OMD, were recruited from the outpatient department of the Institute of Neuroscience, Kolkata (IN-K), a tertiary care centre in eastern India specialising in neurological conditions. (2) Participants for the NT group were recruited using word-of-mouth and supported housing organisations in and around Kolkata. All participants were above 50 years old and resident in India.

To ensure minimal overlap between the OMD and PD groups, patients with motor disorders exhibiting features of atypical parkinsonism, such as progressive supranuclear palsy (PSP), multiple system atrophy (MSA) or secondary parkinsonism (resulting from medications or other underlying conditions), were excluded from the study. For the typically ageing control group, it was ensured that none of the recruited individuals had a diagnosis of PD or any other neurological disorders.

### Measures

All patients in the PD and OMD groups were clinically diagnosed by certified neurologists in IN-K. In the PD group, the severity of symptoms was assessed using the Hoehn and Yahr (H&Y) scale (Hoehn & Yahr, 1967) (see Supplementary Table 1).

Autistic traits were measured using the short form of the Autism Spectrum Quotient (AQ-S) (Hoekstra et al., 2010), consisting of 28 items, each rated on a 4-point scale. Higher total scores indicate the presence of more autistic traits. Respondents were informed that the questionnaire was about their life in general, and not about a specific time window close to the point of response. We opted for the shorter version of the questionnaire based on feedback from pilot testing, which indicated that the original 50-item AQ was inconveniently long for our study participants.

Cognitive ability was measured using the Montreal Cognitive Assessment Scale (MoCA) (Nasreddine et al., 2005). As sleep and GI function are often affected in older individuals, background information on these constructs was collected using (1) items chosen from the Mayo Sleep Questionnaire (Boeve et al., 2011) and (2) a four-item questionnaire measure of GI symptoms (Hjortswang et al., 2006), respectively.

### Procedure

The questionnaires were administered by three researchers, with each set of assessments requiring approximately 45 min to complete. Fifty-six participants were unable to complete the questionnaires independently and were assisted by their respective caregivers. All such instances were noted and modelled within the analyses.

### Statistical analysis

The hypotheses were preregistered on https://aspredicted.org/5YW_YML

Analyses were performed using Jamovi Version 2.2 (The jamovi project, 2022). Descriptive statistics are reported in Table 1.

**Table 1.** Summary of diagnostic group data.

Diagnostic group	N	Sex (male/female, N)	Age (mean ± SD)	AQ total score (mean ± SD)	MoCA total score (mean ± SD)
PD	110	71/39	68.2 ± 8.00	64.85 ± 7.80	16.7 ± 6.08
OMD	110	65/45	62.7 ± 8.08	64.12 ± 7.65	19.0 ± 5.88
NT	110	58/52	63.9 ± 9.34	60.25 ± 8.13	21.6 ± 4.53

To determine whether there was a statistically significant difference in autistic traits between the three groups, a general linear model -based analysis was conducted with AQ as the dependent variable, and group and cognitive ability as predictors. Post hoc comparisons were performed to identify which specific groups differ from each other.

Exploratory correlation analyses were conducted to examine the relationships between:

(a) AQ scores and MoCA scores to test the relationship between autistic traits and cognitive functioning across the whole sample.
(b) AQ and severity of PD (as measured using the H&Y scale) only within PD patients.

In light of known sex differences in AQ (Baron-Cohen et al., 2001) and within PD (Baldereschi et al., 2000), further exploratory analyses (not preregistered) were conducted by including biological sex and age as additional predictors within the linear model.

## Results

### Sample characteristics

From the initial sample of N = 394 individuals, 330 participants were included in the final analyses. A total of n = 64 participants were excluded for not meeting the eligibility criteria (Supplementary Table 1).

The following linear model was fitted to the data:

$$\begin{array}{l} AQ\;\sim \;1+Group\;\left(PD/OMD/NT\right)\\ \;\;\;\;\;\;\;\;\;\;\;\;\;\;\;+Cognitive\;ability\;\left(MoCA\right)\\ \;\;\;\;\;\;\;\;\;\;\;\;\;\;+Reporter\;\left(self/caregiver\right) \end{array}$$

The generalised linear model (GLM) analysis revealed a statistically significant effect of diagnostic group on autistic traits (F(2) = 7.08, p < 0.001, η²p = 0.042). Furthermore, cognitive ability significantly predicted autistic traits (F(1) = 4.32, p < 0.038, η²p = 0.013). No main effect of reporter was observed (F(1, 323) = 0.0715, p = 0.789). In addition, no interaction between diagnosis and reporter was noted (F(2, 323) = 0.9228, p = 0.398).

**Figure 1.**
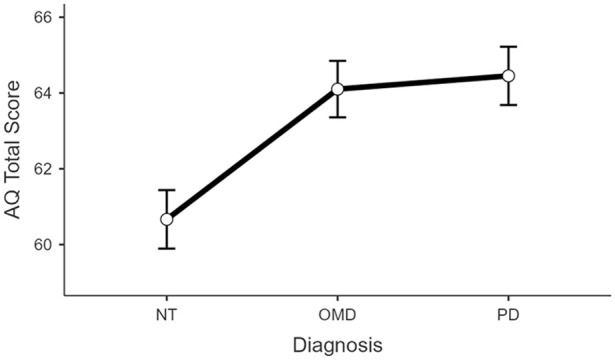
Comparative analysis of total AQ score across three groups – PD, OMD and NT. Error bars represent 1 standard error of mean.

Post hoc pairwise comparisons between the NT group and the OMD group, as well as between the NT group and the PD group, demonstrated significant differences in AQ scores (t = −3.199, df = 326, p = 0.002 and t = −3.371, df = 326, p < 0.001, respectively), with higher scores in the other motor and PD groups compared to NT. However, no significant difference in AQ score was found between the OMD and PD groups (t = −0.327, df = 326, p = 0.744) (Figure 1).

**Figure 2.**
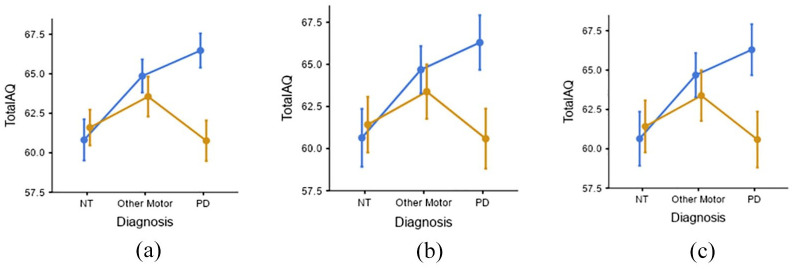
(a), (b), (c): Sex × Diagnosis interaction effect on AQ scores. Error bars depict 1 standard error of mean and are drawn separately for participants filling in the questionnaires in three different languages. (a) Bengali (n = 221), (b) Hindi (n = 67), (c) English (n = 42).

Follow-up exploratory analyses stratifying the sample by biological sex revealed distinctly different patterns of results for the two sexes. The analysis showed a significant main effect of sex (F(1, 315) = 5.0446, p = 0.025, η²p = 0.016). Furthermore, the post hoc tests indicated a significant pairwise difference for males between the PD and NT groups, t(315) = 3.3906, p = 0.012), PD males having significantly higher AQ scores compared to NT males. However, no significant pairwise difference was found between PD and OMD groups (t(315) = 2.5012, p = 0.193). No significant pairwise difference between diagnostic groups was observed among females (Figure 2).

Since the AQ was administered in three languages due to the diverse nature of the sample, we repeated the analyses with language as an additional predictor. Language of administration did not exhibit a statistically significant effect on AQ scores, F(2,315) = 0.2726, p = 0.762, η^2^p = 0.002. The significant sex × diagnosis interaction was consistent across the different languages, providing a degree of internal replication.

The H&Y stage states the severity of motor symptoms based on a scale from 1 to 5, with higher stages indicating more advanced disease progression. A General Linear Model examined the relationship between the H&Y stage and AQ, only within the PD group. The overall model was not statistically significant (F(6, 103) = 1.04, p = 0.403), indicating that the H&Y stage did not significantly predict AQ.

In light of a theoretical suggestion that individuals with PD can be classified into subgroups based on their sleep characteristics (Berg et al., 2021), an exploratory analysis was undertaken to stratify the PD group based on three sleep-related metrics. These metrics included (1) total sleep hours, (2) total number of times waking up at night, and (3) REM sleep behaviour disorder symptoms (sum of items 2, 4, 5, 6, 7, 8 of the Mayo Sleep Questionnaire). For each of these three parameters, a median split was conducted to categorise the sample into data-driven subgroups, and a linear regression analysis was conducted with subgroup, age, and sex as predictors.

There was no difference in autistic symptoms between the PD subgroups defined using any of the sleep parameters, that is, total sleep hours (β = 1.0975, p = 0.454), total number of times waking up at night (β = 0.6843, p = 0.636), and REM sleep behaviour disorder symptoms (β = 2.627, p = 0.086).

A median split of GI symptom scores (median = 160) was carried out for the PD sample to classify its members into two groups (top/bottom half). Linear regression of the AQ with group membership (top/bottom half of the GI symptoms) and sex and age as predictors revealed no effect of group (β = 0.1619, p = 0.914).

For completeness, a comparison of GI and sleep scores for all three groups and a test of their group differences are included in Supplementary Table 4. Groups differed significantly in REM sleep behaviour disorder and GI symptoms.

## Discussion

While several studies have suggested elevated prevalence of parkinsonism in autistic individuals, there are little data on the presence of autistic symptoms in PD. To evaluate from a dimensional perspective the putative overlap between these conditions and its specificity, this study measured autistic traits in a sample of individuals with PD, other motor disorders (OMDs; not PD) and age-matched controls. Elevated autistic traits were observed in both the PD and the OMD groups, compared to the age-matched controls, associating motor disorders in general with greater autistic features. A notable difference between sexes was found, where PD males had greater autistic symptoms compared to those with OMD, who in turn had greater autistic symptoms compared to age-matched male controls. In contrast, no group differences in autistic symptoms were noted between these three diagnostic groups in females. This pattern of results was internally replicated across the three languages in which the instruments were administered (Bengali, Hindi and English) and was not influenced by reporter identity (self or caregiver).

In light of the suggested overlap between autism and parkinsonism, we had expected that PD groups would demonstrate higher autistic-like symptoms compared to both OMD and control groups. However, while both PD and OMD groups were found to demonstrate higher autistic-like symptoms compared to the controls, the difference between the two clinical groups was not significant. The OMD group was chosen to exclude the presence of parkinsonism and PD-like symptoms. Hence, this observation of elevated autistic traits in this group points to a broader possible overlap between motor conditions and autism, rather than a restricted, specific overlap between PD and autism.

These observations admit at least two potential explanations. First, greater autistic features can potentially be driven by greater difficulties with motor control seen in both PD and OMD. Motor control issues can affect eye and facial muscle movements. Altered dynamics of these muscles inevitably impact aspects of social interaction, such as the expression of social signals, which rely heavily on accurate and subtle movements of eyes, faces and body (Vernooij et al., 2016) and can thus lead to difficulties in social communication. Motor difficulties can also contribute to executive dysfunction since motor control is a key component of the implementation of any executive function. Such difficulties in turn could also result in potentially repetitive behaviour. Consider, for example, trying to turn off a tap after washing one’s hands. If difficulties with motor control lead to either too little or too much force when turning off the tap, the individual may need to make several attempts to get the force just right. Such multiple attempts to turn off the tap ‘just right’ can present as repetitive behaviour. However, this account of autistic features arising as a result of greater motor control issues in the PD and OMD groups would predict that greater severity of PD would be associated with greater autistic features. Since there is no evidence from the current dataset to suggest that PD severity (as measured using the H&Y scale) is associated with greater autistic features, we must consider alternative accounts. One alternative account would suggest a broader overlap between the genetic and neural underpinnings of autism and motor disorders, extending beyond PD (Damaj et al., 2015; Giocondo & Curcio, 2017; Mai et al., 2023). According to this account, overlapping genetic factors and/or brain areas can give rise to both higher autistic-like symptoms and the likelihood of developing PD or conditions included within OMD. Beyond the biological insights, these data also present clinical implications. Many older adults with a diagnosis of PD or OMD might not have a formal diagnosis of autism but may present with high autistic features, which often come with specific sensory and/or social needs. It is important to address these needs while designing an appropriate clinical care package for them.

Exploratory analyses revealed an interaction effect between diagnosis and sex on autistic-like symptoms. While males showed the expected pattern of results, with PD associated with the highest level of autistic features, followed by OMD and control groups, no such group differences were noted in females. The sex differences in autistic features were the most prominent for the PD group and were not significant for the other two groups. Two potential explanations are put forward in interpreting these results. First, past studies on both autism and PD show a significantly higher prevalence in males compared to females (Baldereschi et al., 2000; Baron-Cohen et al., 2001). Sex differences are well established within the nigrostriatal dopaminergic pathway, which is affected in PD. Female gonadal factors, specifically estradiol, offer a protective effect, while male gonadal factors either do not provide the same level of protection or may exacerbate the loss of dopaminergic neurons (Gillies et al., 2014). A similar female protective effect, potentially mediated by sex steroids, has also been suggested in autism (Wigdor et al., 2022). An alternative explanation suggests that sociological factors drive females to mask or camouflage their autistic features (Attwood, 2007). Camouflaging or masking is a coping strategy to deal with difficulties during social situations by either hiding behaviour that might be viewed as socially unacceptable or artificially ‘performing’ social behaviour deemed to be more NT. If females in the current sample systematically exhibited more camouflaging or masking behaviours irrespective of diagnostic status, that could explain the lack of significant group differences in females, but not in males.

A previous theoretical model had suggested a distinction between ‘brain-first’ and ‘body-first’ subgroups within PD, where the former is hypothesised to originate in the central nervous system, particularly the brain, and the latter in peripheral tissues, including the GI tract, and thence to progress towards the brain, with each subtype each exhibiting distinct patterns of sleep behaviour (Berg et al., 2021). We explored the implications of this model by stratifying the PD group into two subgroups based on three sleep-related parameters. However, we found no difference in autistic symptoms between these groups. This observation is specific to the current measure of autistic symptoms and sleep behaviour, and does not rule out the possibility of discrete subgroups within PD which can be distinguished from one another on the basis of specific sleep parameters. It is possible that such subgroups exist and differ on phenotypic characteristics other than autistic symptoms. Using a similar approach, we stratified the PD group into two subgroups based on their GI symptoms, which did not show any difference in their autistic symptoms.

Within the full sample, diagnostic group differences in REM sleep behaviour and GI symptoms were observed, with the PD group experiencing the highest level of REM sleep disturbances and GI problems. This result is consistent with previous reports, which suggest that sleep and GI disorders are very common in PD (Fasano et al., 2015; Lees et al., 1988).

While providing initial systematic evidence of autistic traits in PD in relation to OMD and age-matched controls, it is important to consider potential limitations and implications for future research. Autistic symptoms were measured using a questionnaire, which limits the scope of inference. Future studies should ideally include a combination of a questionnaire-based measure along with performance in tasks that tap different domains of the autistic phenotype. A second limitation is the lack of longitudinal data, as it is unclear whether the observed overlap is driven by dynamically evolving motor symptoms within PD. Such a possibility is unlikely, since (1) autistic traits are believed to be largely stable across the adult lifespan (Robinson et al., 2011; Taylor et al., 2017), and (2) we found no evidence for higher autistic features in those with more severe PD. However, a large-scale longitudinal investigation will be necessary to directly test for potential causal links between these constructs. Another limitation of this study is that the OMD group was defined on the basis of clinically verified non-parkinsonian motor impairments. In the absence of genetic profiling, we cannot determine whether some individuals in the PD or OMD groups had known genetic mutations (e.g. PARK2) that have been previously associated with autism. Future studies incorporating genetic profiling could investigate such potential shared genetic factors to explain the current observation of elevated autistic features in both PD and OMD.

Finally, the OMD group is significantly more clinically heterogeneous than the PD group. The crucial common feature in all individuals within the OMD group was that (1) no one had parkinsonian symptoms; (2) everyone had a recognised motor disorder, diagnosed by an expert clinician; and (3) was of comparable age to the PD group. As such, the OMD group, despite its heterogeneity, provides an important test of the generalisability of the suggested overlap between autism and parkinsonism. If the overlap were specific to parkinsonian symptoms and autism and driven by shared factors, then we would expect to see elevated autistic features in PD, but not in the OMD group. However, we found evidence for elevated autistic features within the OMD group as well, suggesting that the putative overlap between autism and PD might extend beyond PD. The small number of individuals within each diagnosis included within the OMD group precluded statistically meaningful subgroup analyses based on specific diagnoses. Future research with adequately powered diagnostic subgroups will be necessary to delineate potential condition-specific associations with autistic traits.

## Conclusion

This study is the first attempt to examine the degree of overlap of autistic traits and PD. The findings suggest that this difference is not exclusive to PD but is also present in OMDs, indicating elevated autistic traits across various motor-related conditions. However, a notable sex difference was observed, males showing a clear pattern of diagnostic group differences in autistic features while females showing no such group differences. This study supports the emerging view of an overlap between autism and PD and extends it to a broader set of motor disorders. It points to the importance of considering autistic features in older adults who may have a diagnosis of PD or a motor disorder while designing clinical support services for them.

## Supplemental Material

sj-docx-1-aut-10.1177_13623613251362267 – Supplemental material for Elevated autistic features in Parkinson’s disease and other motor disorders Supplemental material, sj-docx-1-aut-10.1177_13623613251362267 for Elevated autistic features in Parkinson’s disease and other motor disorders by Ipsita Dey, Swarnima Pathak, Sreerupa Chakrabarty, Matthew K. Belmonte, Supriyo Choudhury, Hrishikesh Kumar and Bhismadev Chakrabarti in Autism
