# Supplementary material for: Elevated autistic features in Parkinson’s disease and other motor disorders
Source: Autism. 2025 Aug 26;29(12):3147–55. doi: 10.1177/13623613251362267 (PMC12618710; doi:10.1177/13623613251362267)
Supplement: sj-docx-1-aut-10.1177_13623613251362267 – Supplemental material for Elevated autistic features in Parkinson’s disease and other motor disorders [file sj-docx-1-aut-10.1177_13623613251362267.docx]

**Supplementary Table 1. Participant Selection Process**

| **Description** | **Count** |
| --- | --- |
| Initial participants | 394 |
| Total Excluded participants | 64 |
| Unavailability of doctor's prescription to verify their diagnosis | 3 |
| Participant dropout during assessment | 4 |
| Presence of atypical parkinsonism or other disorders instead of PD | 24 |
| No confirmed diagnosis made by neurologists | 11 |
| >3 items missing data in the AQ questionnaire | 22 |
| Final sample size (N) | 330 |
| Sample size per diagnostic group (n) | 110 |

**Supplementary Table 2: Severity Levels of PD using Hoehn and Yahr Stage Scale**

| **Hoehn and Yahr Stages** | **Characteristics** | **n(%)** |
| --- | --- | --- |
| Stage 1 | Unilateral involvement only. | 17(15.45) |
| Stage 1.5 | Unilateral and axial involvement. | 5(4.55) |
| Stage 2 | Bilateral involvement without impairment of balance. | 39(35.45) |
| Stage 2.5 | Mild bilateral involvement with recovery on retropulsion (pull) test. | 20(18.18) |
| Stage 3 | Mild to moderate bilateral involvement, some postural instability but physically independent. | 18(16.36) |
| Stage 4 | Severe disability, still able to walk and to stand unassisted. | 6(5.45) |
| Stage 5 | Wheelchair bound or bedridden unless aided. | 5(4.55) |
| **Total** |  | **110** |

**Supplementary Table 3: Characteristics of the Other Motor Group participants (N=110)**

| **Condition Name** | **Frequency** |
| --- | --- |
| Essential Tremor | 10 |
| Hemifacial Spasm | 9 |
| SCA 12 | 9 |
| Meige's syndrome | 8 |
| Blepharospasm | 6 |
| Cervical Dystonia | 6 |
| Oromandibular Dystonia (OMD) | 5 |
| Segmental Dystonia | 5 |
| Writer's cramp | 5 |
| Dystonic Tremor | 4 |
| Huntington's disease | 4 |
| Tardive dyskinesia | 4 |
| Left Hemifacial spasm | 3 |
| Right Hemifacial Spasm | 3 |
| Facial Spasm | 2 |
| Focal Task Specific Dystonia | 2 |
| Generalized Dystonia | 2 |
| Ataxia | 1 |
| Basal Ganglia Infarct | 1 |
| Cerebral Ataxia | 1 |
| Cervical Myelopathy | 1 |
| Choreo Dystonic movements | 1 |
| Dystonia, Ataxia | 1 |
| Essential tremor/ Dystonic tremor | 1 |
| Familial Adult-Onset Chorea | 1 |
| Focal dystonia | 1 |
| Left hemifacial spasm | 1 |
| Lingual Dystonia | 1 |
| Myasthenia Gravis | 1 |
| Multiple Sclerosis | 1 |
| Oromandibular Dyskinesia | 1 |
| Postural Tremor | 1 |
| Preoral Dyskinesia | 1 |
| Progressive Muscular Atrophy | 1 |
| Progressive proximal muscle weakness | 1 |
| SCA 2 | 1 |
| Spastic Paraparesis | 1 |
| Transverse Myelitis | 1 |
| Trigeminal neuralgia | 1 |
| Truncal dystonia | 1 |
| **Total** | **110** |

**Supplementary Table 4 (a): Descriptive Statistics and Group Difference (N=330)**

|  | **PD** | **OMD** | **NT** | **Group difference statistics** | |
| --- | --- | --- | --- | --- | --- |
|  | **Mean ±SD** | **Mean ±SD** | **Mean ±SD** | **F(2, 327)** | **η2** |
| GI Symptoms | 165.9 ± 78.70 | 115.6 ± 57.79 | 92.0 ± 65.0 | 34.2*** | 0.173 |
| Sleep |  |  |  |  |  |
| Number of times waking up at night | 0.823 ± 1.12 | 1.46 ± 1.05 | 0.691 ± 1.04 | 16.1*** | 0.087 |
| REM Sleep behaviour | 166 ± 78.7 | 116 ± 57.8 | 92.0 ± 65.0 | 7.99*** | 0.047 |
|  |  |  |  | **F(2, 321)** | **η2** |
| Total sleep hours | 6.99 ± 1.90 | 7.05 ± 1.44 | 6.97 ± 1.95 | 0.143 | 0.001 |

***p < .001

*Note: Six data points were removed from the dataset from ‘Total sleep hours’ due to implausible values (0.0 hours), resulting in a final sample size of 324 for the analysis.*

**Supplementary Table 4 (b): Post-hoc Test (N=330)**

| **Variable** | **Comparison** | **Mean Difference** | **SE** | **t** | **p** | **Adjusted p** |
| --- | --- | --- | --- | --- | --- | --- |
| GI Symptoms | PD vs OMD | 50.3 | 9.13 | 5.51 | < .001 | < .001 |
|  | PD vs NT | 73.9 | 9.13 | 8.09 | < .001 | < .001 |
|  | OMD vs NT | -23.6 | 9.13 | -2.59 | 0.030 | 0.030 |
| Number of times waking up | PD vs OMD | -0.635 | 0.144 | -4.4 | < .001 | < .001 |
|  | PD vs NT | 0.132 | 0.144 | 0.91 | 0.361 | 1.000 |
|  | OMD vs NT | 0.766 | 0.144 | 5.32 | < .001 | < .001 |
| REM Sleep Behaviour | PD vs OMD | 50.0 | 9.13 | 5.48 | < .001 | < .001 |
|  | PD vs NT | 73.9 | 9.13 | 8.09 | < .001 | < .001 |
|  | OMD vs NT | -23.6 | 9.13 | -2.59 | 0.030 | 0.030 |
